# A Multi-Disciplinary Approach to Assess the Welfare Impacts of a New Virtual Fencing Technology

**DOI:** 10.3389/fvets.2021.637709

**Published:** 2021-02-23

**Authors:** Caroline Lee, Dana L. M. Campbell

**Affiliations:** CSIRO, Agriculture and Food, FD McMaster Laboratory, Armidale, NSW, Australia

**Keywords:** animal welfare, behavior, cattle, cortisol, cognition, stress, sheep

## Abstract

Virtual fencing involving the application of audio cues and electrical stimuli is being commercially developed for cattle. Virtual fencing has the potential to improve productivity through optimized pasture management and utilization by grazing animals. The application of virtual fencing initiates public concern for the potential welfare impacts on animals due the aversive nature of using an electrical stimulus. It is therefore important to provide welfare assurance of the impacts of virtual fencing on livestock. In this paper, we provide an overview of the welfare assessment and validation stages for virtual fencing which could be applied to other new technologies utilizing novel systems. An understanding of stress measures and their suitability for use in specific contexts is discussed, including the use of glucocorticoids to measure both acute and chronic stress, and behavioral responses and patterns to indicate welfare states. The importance of individual differences in relation to learning and cognition are also highlighted. Together, this multi-disciplinary approach to welfare assessment provides a tool kit that may be applied for welfare assurance of some new technologies and systems for farm animals.

## Introduction

Utilization of livestock by humans has depended on the capacity of animals to adapt to new farming technologies like herding, milking and harvesting of fiber and eggs. Further advances in husbandry systems and management technologies, such as virtual fencing, intensive housing, and automated milking parlors have increased complexity of the environment farmed animals must learn to engage with. Adaptation to new systems involves cognitive evaluation of environmental stimuli which influences the stress response and subsequent adaptation (1). Assessment of the welfare impacts of implementing new technologies and systems is needed to ensure welfare is acceptable.

Virtual fencing involves the containment of animals without the use of a physical fence by using signals from a device that is attached via a neckband. Using GPS technology to monitor animal movement and behavior, an audio cue signal warns the animal that it is approaching the virtual boundary, and this is followed by an electrical pulse only if the animal does not respond to the audio cue (2–5). The device applies an electrical pulse sequence in the kilovolt range with an intensity that is lower in energy than an electric fence (6). Successful learning occurs when the animal responds to the audio cue to stay within the boundary and avoids receiving the electrical pulse. On some occasions, an animal may cross the virtual fence line and no stimuli are applied if the animal turns and moves toward the inclusion zone to encourage movement back within the boundary (7). In a 44 day study, the virtual fence was 99.8% effective at preventing cattle accessing a sapling regeneration area (8). As the virtual fencing is not 100% effective at containing livestock, fixed fencing should be used for external boundaries and the virtual fence should only be used for internal fencing to reduce the risks of animals accessing roads or public areas. When the virtual fence location is moved, both cattle (9) and sheep (10) enter the new paddock area within hours, demonstrating that they learn to respond to the audio cue and not the location that cues are given, this has important implications for pasture management and strip grazing applications. Virtual fencing has the potential to transform livestock (cattle and sheep) farming (11, 12) by optimizing pasture management, managing weeds in mixed farming systems, maintaining separation to prevent fighting (13) and protecting environmentally sensitive areas (7, 8). Removal of physical fencing also has the potential to benefit wildlife conservation (14). The virtual fencing technology is being commercialized by Agersens (Melbourne, VIC, Australia) and a product for cattle (eShepherd®) will be released imminently. However, the use of an aversive electrical pulse generates concern from the public in relation to animal welfare impacts and science-based evidence to provide welfare assurance is required (15).

Assessing the welfare impacts of virtual fencing in livestock, requires a multi-disciplinary approach to account for the complexity of the animal interacting with and learning about a new technology autonomously, while in a field situation. Consideration of physiological indicators of acute and chronic stress, behavioral responses and patterns, cognition, associative learning, and social learning are all necessary. This review will discuss and highlight the challenges of providing a comprehensive assessment of animal welfare in relation to a new livestock farming technology. The findings from studies investigating the effects of virtual fencing on measures of acute and chronic stress and animal learning will be considered in relation to the welfare implications of this technology and ethical assurance for stakeholders.

## Stages of Learning

**Figure 1:**
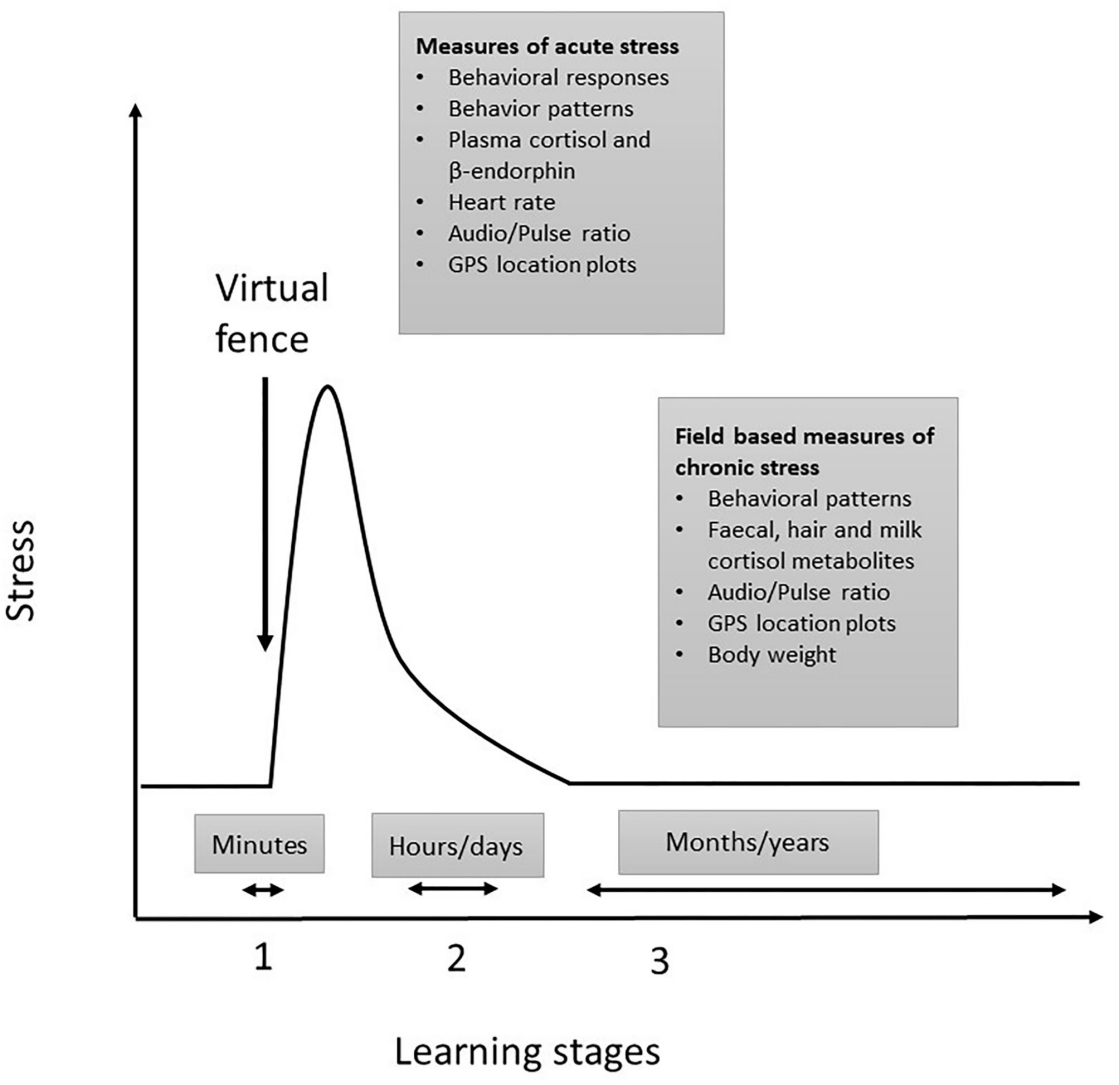
Proposed relationship between learning the virtual fence and stress in livestock. Stage 1 represents the acute phase of learning when animals cannot avoid receiving the electrical pulse and this induces an acute stress response. Stage 2 describes animals adapting to the virtual fence. Stage 3 is where associative learning has occurred, and animals can control their interaction with the fence and avoid receiving the electrical pulse. Relevant measures for acute and chronic stress responses are listed within the text boxes.

We propose that the stress responses of livestock differ in relation to the stages of virtual fence learning. The first stage of virtual fence learning requires the animal to experience both the audio cue and the aversive electrical pulse to enable subsequent associative learning to occur (Figure 1). In this initial period, animals cannot avoid receiving the electrical pulse [but see (16) for impacts of social facilitation on behavioral responses], and so the relative aversiveness of the electrical pulse will determine the intensity and duration of the acute stress response (17, 18). Following this, there is a period of adaptation (stage 2) to the virtual fencing system where animals may be in an aroused state until they have learnt to respond to the audio cue and are able to avoid receiving the electrical pulse. Finally, stage 3 is where learning has occurred, and the animals are able to predict and control their interaction with the fence. In this final stage, the fence position is indicated by an audio cue and may shift location. Thus, cattle need to rely on responding correctly to the sound to avoid the electrical pulse without any accompanying visual information which contrasts with being able to see the visual barrier of a standard (electric) fence. For each of these stages, the timelines vary, and different measures are relevant. The acute stress measures are applicable to the initial learning period which typically has a duration of minutes and the chronic stress measures are applicable to later stages of learning. The stage 2 period of adaptation may last for a few hours up to a few days, but stage 3 implementation of a virtual fence could be weeks, months, or potentially years. For welfare to be assured, the effects of virtual fencing on key measures during stage 1 and 2 should be minimal and in stage 3, should not differ from control treatments or normal baseline measures.

## Physiological Indicators of Stress

Physiological measures that have been applied to assess acute stress responses to virtual fencing include circulating plasma concentrations of stress hormones, such as cortisol and β-endorphin, measures of heart rate and body temperature increases that may indicate stress induced hyperthermia. To ensure that the stress response measured is due to the exposure to the virtual fencing stimuli themselves and not due to other factors, it is important to have in place a robust experimental design including a control treatment, minimal handling and/or habituation. Controlled studies are necessary where the stimuli are manually applied to account for potential variation in self-exposure to stimuli among individual animals. Other physiological measures that could be applied to the assessment of stress include infrared thermography (19), functional near-infrared spectroscopy (20), and electroencephalography (21).

### Concentrations of Stress Hormones

The hypothalamic-pituitary-adrenal (HPA) axis is activated in response to a stressor with a clear relationship between stressor intensity and duration and the HPA axis activation. Stress hormones including glucocorticoids (e.g., cortisol) and opioids (e.g., β-endorphin) are released as part of a cascade when stressors are perceived by the brain (22, 23). As handling itself is stress inducing, blood samples should be collected within 2–3 mins of restraint, before the adrenal cortex has been activated (23). An alternative is to habituate animals to handling prior to the study and include a control treatment to show that cortisol responses are not elevated by handling itself (18). These considerations for the measurement of acute stress hormone concentrations in the context of controlled experimental studies enables comparisons to be made between treatments. To demonstrate this, plasma cortisol and β-endorphin concentrations were assessed in beef cattle receiving an electrical pulse compared with a range of common husbandry procedures and this showed that the stress response to an electrical pulse was not different to being restrained in a crush (17). In a similar comparison study with sheep, a mild cortisol response to an electrical pulse was shown and this was similar to hearing a barking dog (18) and sheep did not differ in their cortisol responses to the audio cue once they had successfully learnt the virtual fence (24). Overall, these results indicate that while the electrical pulse is aversive, it is not more stressful than common handling procedures in both sheep and cattle.

While plasma stress hormones are good measures of arousal in short-term controlled experiments, they are less suitable for measurement of chronic stress in field-based studies. Plasma cortisol is affected by the sampling procedure itself and levels usually decline after the acute response so are not very informative for states of chronic stress (23). In addition, chronic stress can modify the responsiveness of the HPA axis, with a range of effects, including both an increase in the responsiveness (25) and a decrease in the sensitivity of the HPA axis following negative events (26, 27). Measurement of cortisol metabolites in feces, hair or milk are more stable and therefore are practical options for assessment of chronic stress in longer-term field studies (28, 29). When virtual fencing was compared with conventional electric fencing, fecal cortisol metabolites did not differ over a 4-week period, indicating that there were no differences in stress responses over that period between fencing groups although the metabolites did reduce across time (6). Similar findings were reported in dairy cows, with no differences between virtual and conventional electric fencing on milk cortisol concentrations for a 5-day period, however longer-term assessment is needed (30).

### Heart Rate

Other physiological measures of stress include heart rate and heart rate variability (HRV), which indicate a change in cardiac function and provide an early indicator of stress responses (31, 32). A heart rate device is strapped around the girth area of an animal and the area is shaved to enable close contact of the electrodes with the skin. While heart rate and HRV measures are feasible in controlled experimental contexts, they are not yet practical for longer-term field deployment mainly due to issues with attachment (33). However, progress in developing heart rate measures in cattle with high accuracy for use in the field is occurring (34). In addition, heart rate is affected by locomotion (35) so care should be taken when designing studies using this measure. In the cattle study that measured stress hormone responses to the electrical pulse and common husbandry procedures, a second experiment assessed heart rate responses and found that they did not differ between any treatments which confirmed the stress hormone findings (17).

### Stress-Induced Hyperthermia

Stress-induced hyperthermia (SIH), a rapid increase in core body temperature due to exposure to a stressor, can be used to measure acute stress responses (31). Small temperature loggers collect data and are placed in the vagina or rectum of the animal (36, 37). SIH has been demonstrated in sheep during shearing (38), isolation (39) and when anxious (40–42), and in cattle during handling (43) or when anxious (44). However, SIH was not observed in sheep exposed to virtual fencing stimuli either in a controlled experiment (18) or in the field (45). This may have been due to the stimuli intensity or duration not being sufficiently aversive to induce a stress response. Thus, while SIH has been an accurate and practical measure of stress response deployed in both experimental and field contexts, its relevance to welfare assessment of virtual fencing is uncertain. The short-lived duration (<1 s) of electrical pulse exposure and the substantial variation in self-exposure both within and among individuals may limit interpretations of this measure.

### Body Weight

A coarser indicator of welfare is changes in body weight over time where a lower body weight gain may be indicative of a welfare issue (46). But this can be influenced by many factors, including feed and water availability, health, climate, and physiological status and thus may be most informative if paired with other simultaneous welfare measures. If used as part of a controlled study, it may be a valuable measure but to date has not provided consistent indications of welfare impacts of virtual fencing (6).

## Behavioral Indicators of Stress

### Behavioral Responses

Immediate behavioral responses specifically to the stimuli provide an indicator of their aversiveness and effectiveness. The audio cue alone should be benign, eliciting no specific reaction beyond ear movement until it has been associated with the electrical pulse. This has been observed when cattle first hear the audio tone (4) although sheep appear more sensitive to the audio signal with first exposure (5, 47). With the electrical pulse, it needs to be aversive enough that it deters the animal, but extreme and extended behavioral responses such as leaping forward, vocalizing, and jumping are undesirable and may reduce an animal's learning ability while in such a heightened state (2). A stimulus that is highly aversive is inappropriate to use (2, 5), and in the case of developing the virtual fencing pre-commercial prototypes in cattle, alternative pulse durations and intensities were tested to optimize the electrical pulse (4). Additionally, poor or inconsistent pulse delivery may result in animals that show a minimal behavioral response (e.g., head tossing or turning in cattle) to both the audio and pulse stimuli whilst continuing to move past the virtual fence (8). Ultimately, this could have welfare consequences if they attract others to follow, thus increasing stimuli delivery for some individuals, potentially causing confusion, frustration and stress. Individual variation in skin sensitivity and pain perception may increase the aversiveness for some animals with some evidence of variation in dairy cows (48, 49) but further investigation into this is required.

### Behavioral Patterns

Monitoring of behavioral patterns of the individual and the herd are a practical indicator of welfare to deploy in field studies. Although precise behavioral patterns vary among individuals and herds, and within herds relative to season or across age (50), deviations from what is expected to be “normal” for that species may be indicative of chronic stress. With the availability of increasing numbers of off-the-shelf monitoring products such as IceQubes® and Moomonitors® (51) for cattle (6) and HOBO's for sheep (45) that have relatively long battery life, long-term monitoring of cattle and sheep behavior is now possible. Disturbances in normal behavioral patterns over time may indicate that welfare is not optimal, for example, lying time has been demonstrated to indicate comfort of lying surfaces in cattle (52). In a study using pre-commercial eShepherd® prototype devices for cattle where virtual fences were moved at regular intervals for a 22-day period, behavioral time budget changes were minor (9). Similarly, minimal behavioral pattern changes were reported in a longer 4-week study using the virtual fencing system in beef cattle (6) or for a shorter 5-day period with dairy cows (30). Further assessments of behavioral time budgets over longer periods would be recommended in future research to confirm these findings.

### GPS Location Data

GPS location of individual animals can be used to assess if animals show a lack of understanding of where the virtual fence is located as evidenced by thigmotaxis, a tendency to move toward physical contact, such as an increased following of fixed fences. Rodents show thigmotaxis when anxious (53) and it is thought to be a protection against predators (54). No evidence has been reported of thigmotaxis in any of the virtual fencing studies using the Agersens system (eShepherd®) and manual dog collars. All GPS plots to date of sheep and cattle locations in the presence of a virtual fence indicate usage of all paddock areas including those immediately in front of the virtual barrier (6–10). Interpretation of GPS data showing spatial distribution of animals should consider the uniformity of the paddock and position of preferred resources as these will influence the time animals spend in certain areas.

## Cognitive Measures of Welfare

### Associative Learning

The ability of animals to predict and control their situation in the long term is strongly related to welfare outcomes (55). Consideration of the impact of sudden changes to predictable routines such as feeding times, and regrouping can have negative impacts (56). As proposed in a welfare assessment framework of virtual fencing (57), once animals learn the association between the audio and electrical stimuli, the cues are both predictable (the audio cue always precedes the electrical pulse) and controllable (animals can choose to avoid the electrical pulse by stopping or turning), thus minimizing negative welfare impacts. Indeed, cattle learn rapidly after an average of 2.5 interactions with the virtual fence before responding to the audio cue alone (6). This hypothesis was tested Kearton et al. (24) in a study that assessed the influence of controllability on stress responses to virtual fencing stimuli. Sheep that had learned to predict and control receiving the electrical pulse through their behavioral responses, did not differ in their cortisol, core body temperature and behavioral responses compared with a control treatment that did not receive any cues. This shows that the sheep perceived the audio cue as benign once they had successfully learnt.

Inclusion of a measure that indicates learning of the virtual fence such as the relative proportions of audio and electrical pulse cues is of value for welfare assessment. This could be used to ensure all animals are learning and have reached set thresholds within a certain number of interactions with the virtual fence. Additionally, it would allow confirmation that all animals being managed by the system have successfully learnt to respond to the audio cue so that it is both predictable and controllable. Identification of animals that are not learning (as indicated by an audio cue always being followed by an electrical pulse) may indicate a learning or equipment failure and providing an alert will enable the animal to be checked and if necessary, removed from the virtually fenced paddock.

### Social Learning

Livestock are social animals that are typically managed in groups forming dominance relationships and social networks (58–60). Associative learning of the virtual fence occurs more rapidly when applied to a group of cattle (7–9, 61) or sheep (10, 47) than when applied to individuals (3, 4). It is likely that the social attraction to remain with the group provides encouragement to respond by turning and re-joining the herd or flock. Previous experience can also affect learning of the virtual fencing stimuli, with pre-exposure to an electric fence in dairy heifers resulting in more rapid associative learning (62). Recently, social facilitation of virtual fence learning was reported in cattle (16) with animals responding when others interacted with the fence. Social influences on the effectiveness of the virtual fence were also shown in sheep, with collaring two thirds of the group with virtual fence collars being equally as effective at containing sheep as having all animals collared (45). More research is needed to understand social learning aspects of virtual fencing, particularly in larger, commercially relevant group sizes.

## Other Considerations

With the identification of distinct personalities (63) and coping styles in sheep (64) and temperament in cattle (65), consideration of individual differences is recommended in evaluating welfare impacts of management practices and new technologies such as virtual fencing. In addition, further research to investigate application of virtual fencing to different stock classes such as cows and calves or ewes and lambs and the impact on animal welfare is needed.

## Conclusions

Welfare assessment of a virtual fencing system requires consideration of the nature of the stress response during the different stages of learning and adaptation to the system. A multi-disciplinary approach applied to assess both acute and chronic stress is needed that also accounts for individual differences in cognition, physiology and behavioral responses. Of importance is the assessment of the chronic stress measures as the acute stress response is short lived and animals quickly adapt. Welfare assessment and validation that focusses on the longer-term impacts across different situations is needed for welfare assurance of new technologies and systems. Application of a range of measures over the short and longer term, have confirmed that welfare impacts of virtual fencing on cattle and sheep are minimal. Further studies to assess the impacts over even longer periods are recommended to confirm these findings in a commercial setting.

## Data Availability Statement

The original contributions presented in the study are included in the article/supplementary material, further inquiries can be directed to the corresponding author/s.

## Author Contributions

CL prepared the first draft and edited the manuscript. DC contributed to the content, wrote parts, and edited the manuscript. All authors contributed to the article and approved the submitted version.

## Conflict of Interest

The authors declare that the research was conducted in the absence of any commercial or financial relationships that could be construed as a potential conflict of interest.
